# Endothelin-1 Exhibiting Pro-Nociceptive and Pro-Peristaltic Activities Is Increased in Peritoneal Carcinomatosis

**DOI:** 10.3389/fpain.2021.613187

**Published:** 2021-02-11

**Authors:** Céline Greco, Lilian Basso, Cléo Désormeaux, Audren Fournel, Benedicte Demuynck, Leila Lafendi, Sylvie Chapiro, Antoinette Lemoine, Ying-Ying Zhu, Claude Knauf, Nicolas Cenac, Claude Boucheix, Gilles Dietrich

**Affiliations:** ^1^UMR-S935, INSERM, Univ. Paris-Sud, Université Paris Saclay, Villejuif, France; ^2^Department of Pain Management and Palliative Care, Necker-Enfants Malades Hospital, AP-HP, Paris, France; ^3^IRSD, Université de Toulouse, INSERM, INRA, ENVT, UPS, Toulouse, France; ^4^Department of Oncology, Montereau-Fault-Yonne Hospital, Montereau, France; ^5^Department of Medical Biology and Physiology, Montereau-Fault-Yonne Hospital, Montereau, France; ^6^Department of Palliative Care, Paul Brousse Hospital, AP-HP, Villejuif, France; ^7^UMR-S1093, INSERM, Univ. Paris-Sud, Université Paris Saclay, Villejuif, France; ^8^Department of Biochemistry, Paul Brousse Hospital, AP-HP, Villejuif, France

**Keywords:** endothelin, peritoneal carcinomatosis, visceral pain, digestive tract tumor, ovarian cancer, peristalsis

## Abstract

**Background:** Peritoneal carcinomatosis often results in alterations in intestinal peristalsis and recurrent abdominal pain. Pain management in these patients is often unsatisfactory. This study aimed to investigate whether endothelin-1 (EDN1) was involved in pain mediation in peritoneal carcinomatosis, and thus whether the EDN1 pathway could be a new therapeutic target for peritoneal carcinomatosis-associated pain.

**Methods:** EDN1 plasma levels and abdominal pain severity were assessed in patients with abdominal tumors, with or without peritoneal carcinomatosis, and in healthy donors. The effects of EDN1 on the visceromotor response to colorectal distension, and on colonic contractions were then examined in mice, and the mechanism of action of EDN1 was then investigated by measuring the impact of EDN1 exposure on calcium mobilization in cultured neurons. Inhibition studies were also performed to determine if the effects of EDN1 exposure could be reversed by EDN1-specific receptor antagonists.

**Results:** A positive correlation between EDN1 plasma levels and abdominal pain was identified in patients with peritoneal carcinomatosis. EDN1 exposure increased visceral sensitivity and the amplitude of colonic contractions in mice and induced calcium mobilization by direct binding to its receptors on sensory neurons. The effects of EDN1 were inhibited by antagonists of the EDN1 receptors.

**Conclusions:** This preliminary study, using data from patients with peritoneal carcinomatosis combined with data from experiments performed in mice, suggests that EDN1 may play a key role mediating pain in peritoneal carcinomatosis. Our findings suggest that antagonists of the EDN1 receptors might be beneficial in the management of pain in patients with peritoneal carcinomatosis.

## Introduction

Peritoneal carcinomatosis (PC) is frequently observed in patients with ovarian or digestive tract tumors. Most patients with PC develop symptoms including intestinal colic, continuous and diffuse abdominal pain, and present abnormal peristalsis associated with nausea and vomiting. Management of the abdominal pain in patients with PC has improved over recent years with the use of combined treatments involving analgesics (opioids), antiemetics, and antisecretory agents [1]. However, for many patients, the level of pain relief achieved with these therapies remains unsatisfactory [2]. New antinociceptive strategies, based on new therapeutic targets, are therefore needed to treat pain in patients with PC.

Endothelin-1 (EDN1), a peptide secreted by endothelial cells [3], has been shown to play key roles in the regulation of smooth muscle fiber contractions and in the mediation of visceral pain [4, 5]. In humans, the EDN1 peptide is derived from a 212-amino acid precursor (prepro-EDN1). This precursor is first cleaved by an endopeptidase to form a biologically inactive 38-amino acid precursor (big-EDN1), and then cleaved further—either by endothelin converting enzymes 1 and 2, or by a chymase—to form EDN1 [6]. Endothelin acts by binding to two G-protein-coupled receptors, endothelin receptor type A (EDNRA), and endothelin receptor type B (EDNRB), located in the gastrointestinal wall [7]. Activation of these EDNRs in peripheral tissues results in the mobilization of intracellular calcium, and has been shown to induce pain in mice [8]. In addition, several previous studies have indicated that EDN1 is involved in the mediation of visceral pain in a number of diseases, including cancer [4, 9–12].

The aim of this study was to investigate whether EDN1 plays a pro-nociceptive role in the mediation of visceral pain in PC, and therefore determine if inhibition of the EDN1 signaling pathway could provide a potential new therapeutic target for the treatment of visceral pain in patients with digestive tract tumors associated with PC. Thus, we first conducted a preliminary study to assess whether there was a correlation between plasma levels of EDN1 and the intensity of abdominal pain in patients with PC. We then evaluated the effects of EDN1 on visceral sensitivity and colonic peristalsis in mice, and investigated the mechanism of activation of EDNRs in dorsal root ganglia (DRG) neurons. We also studied whether the effects of EDN1 in mice could be reversed by exposure to EDNRs antagonists.

## Materials and Methods

### Materials

Synthetic EDN1 peptide was purchased from the American Peptide Company (Sunnyvale, CA). Atrasentan hydrochloride (A-1467267; ATRA), an EDNRA antagonist, and bosentan (BOS), an antagonist of both EDNRA and EDNRB, were purchased from MedChem Express (Monmouth Junction, NJ) and Selleckchem (Houston, TX), respectively.

### Quantification of Endothelin-1 in Plasma Samples and Visceral Pain Assessments in Patients and Healthy Donors

Blood samples (5 mL) were obtained from patients mainly with abdominal primary tumors (colon, small intestine, pancreas, stomach ovary, or kidney), with or without PC, and from healthy donors. Samples were collected in tubes containing EDTA and immediately centrifuged at 2,000 g for 15 min at 4°C. Plasma samples were then recovered, aliquoted, and frozen at −20°C until use. EDN1 levels in the plasma were quantified using the QuantiGlo chemiluminescent sandwich ELISA kit (R&D Systems, Inc., Minneapolis, MN).

Abdominal pain was monitored at the time of the blood collection using the Numeric Pain Rating Scale (NPRS): patients rated their current level of pain on a scale of 0–10, with a score of 0 indicating “no pain” and a score of 10 indicating “the worst pain imaginable.”

Written informed consent was obtained from each patient and healthy donor prior to inclusion in the study. Quantification EDN1 levels in the plasma samples was approved by the local ethics committee of the Sud-Seine et Marne Hospital (05/12/2015). The study protocol conformed to the ethical guidelines of the 1975 Declaration of Helsinki, and its subsequent amendments, as reflected by its a priori approval by the institution's human research committee.

### Electromyographic Assessments of the Visceromotor Response to Colorectal Distension in Mice

Colorectal distension was performed as already described [13, 14] to measure the effect of EDN1 on visceral sensitivity in 8 week-old C57Bl/6 male mice. Briefly, two electrodes (Bioflex insulated wire AS631; Cooner Wire, Chatsworth, CA) were inserted into the abdominal oblique muscles. 3 days later, the mice received an intraperitoneal injection of 200 μL of either a vehicle or EDN1 (100 μg.kg^−1^). The electromyographic activity of the abdominal muscles was recorded 30 min after the injection by connecting the electrodes to an electromyogram acquisition system (PowerLab, ADInstruments Inc., Colorado Springs, CO), *via* a high performance differential amplifier (Bio Amp, ADInstruments). Visceromotor responses were analyzed using the Chart 5 software (ADInstruments). Colorectal distension was achieved by inserting a balloon catheter (diameter: 10.5 mm; Fogarty catheter for arterial embolectomy, 4F; Edwards Lifesciences, Nijmegen, Netherlands) into the colon, 5 mm from the anus. The balloon catheter was then progressively inflated in stepwise increments of 15 mm Hg: 10-s distensions were performed with pressures of 15, 30, 45, and 60 mm Hg and 5-min rest intervals between each distention. The same procedure was then used to analyze colorectal distension in the presence of EDNR antagonists: 30 min before the injection with EDN1, the mice received intraperitoneal injections of 10 mg.kg^−1^ of either ATRA or BOS. All procedures were performed in accordance with the Guide for the Care and Use of Laboratory Animals of the European Council and were approved by the US006/CREFE Animal Care and Ethics Committee (CEEA-122; application number APAFIS #7762- CE2016112509278235V2).

### *Ex vivo* Measurements of Colonic Contractions in Mice

Proximal colon segments (10 mm in length) were obtained from C57Bl/6 mice treated with EDN1 and/or BOS as described above. The segments were incubated in an oxygenated Krebs-Ringer bicarbonate/glucose buffer (pH 7.4) for 30 min prior to being attached to an isotonic transducer (Lever Transducer, B40 type 373, Hugo Sachs Elektronik) and immersed in tubes containing 25 mL of Krebs-Ringer buffer at 37°C. The load applied to the lever was 2 g (20 mN). Isotonic contractions were then recorded for 10 min using the basic data acquisition software (BDAS; Hugo Sachs Elektronik). Average colonic contraction amplitudes were calculated from recordings taken over 10-s intervals and the data collected were then normalized to obtain the average amplitude of the basal contractions, as described previously [15].

### Isolation of Dorsal Root Ganglia Neurons for Calcium Imaging Assays

DRG were collected from C57Bl/6 mice, washed with Hanks Balanced Salt Solution (HBSS, Thermo Fisher Scientific, Waltham, Massachusetts, USA), and incubated in HBSS containing 27 μg.mL^−1^ papain (Sigma Aldrich, Missouri, USA) and L-Cysteine (pH 7.4, Sigma Aldrich, Missouri, USA) for 10 min at 37°C. The DRG were then rinsed in Leibovitz's L-15 Medium (Thermo Fisher Scientific, Waltham, Massachusetts, USA) containing 1% penicillin/streptomycin and 10% Fetal Bovine Serum (FBS) (Thermo Fisher Scientific, Waltham, Massachusetts, USA) and digested twice with 4 mg.mL^−1^ dispase II (Sigma) and 1 mg.mL^−1^ collagenase type I (Sigma) for 5 min at 37°C. Enzyme activity was then neutralized by adding L-15 buffer and the neurons were mechanically dissociated. After centrifugation at 600 g for 5 min, neurons were plated into an 8-well Nunc™ Lab-Tek™ II CC2™ Chamber Slide System (Thermo Fisher Scientific, Waltham, Massachusetts, USA) and cultured in Dulbecco's Modified Eagle Medium (DMEM) containing 2.5% FBS, 1% penicillin/streptomycin and 10 μM of a cocktail of mitosis inhibitors including cytosine-B-arabinofuranoside, F-uridine, and uridine (Sigma) for 3 days before being used for calcium imaging assays.

### Calcium Imaging

Before calcium imaging, the neurons were incubated with HBSS containing 20 mM Hepes, 1 mM fluo-4 acetoxymethyl (AM) ester (Thermo Fisher) and 0.02% pluronic F-17 for 30 min at 37°C, followed by an additional incubation for 30 min at room temperature. The medium was then discarded and replaced by HBSS containing 1.26 mM CaCl_2_. Neurons were then incubated with either 10 μM BOS in 0.001% DMSO HBSS or a vehicle for 5 min before adding increasing amounts of EDN1. Neurons were imaged using an inverted microscope (Zeiss) and a 10x 0.5 NA objective. Images were acquired using a CCD camera (Zeiss) and Zen software (Zeiss). Acquisition parameters were kept constant during each experiment. Kinetic analysis was performed using 80 recordings (one per second). Baseline fluorescence was determined for 0–5 s, and then fluorescence was measured from the 6 to 60 s after the neurons were incubated with the drugs or control. At 60 s 50 mM KCl was added to the cells in order to allow discrimination between neurons and glial cells. Variations in fluorescence intensity measured in each neuron were identified using Image J software. The percentage of responding neurons was calculated as the number of DRG neurons in which EDN1 induced calcium mobilization, relative to the total number of neurons identified by their ability to respond to 50 mM KCl. A neuron was defined as responding when the ratio between the highest fluorescence measured between 6 and 60 s and the baseline measured between 0 and 5 s was higher than 1.

### Statistical Analyses

Results are expressed as the mean ± the standard error of the mean (SEM). Statistical analyses of intergroup comparisons were performed using the nonparametric Kruskal-Wallis test combined with a *post-hoc* Dunn's multiple comparison test using the Prism 5.0 software (GraphPad Software Inc., La Jolla, CA). A two-way repeated-measures analysis of variance (ANOVA) was used to compare two independent treatments over a range of concentrations. The strength of the correlation between abdominal pain intensity and EDN1 plasma concentration was evaluated by using the nonparametric Spearman's correlation coefficient.

## Results

### Study Participants

**Figure 1:**
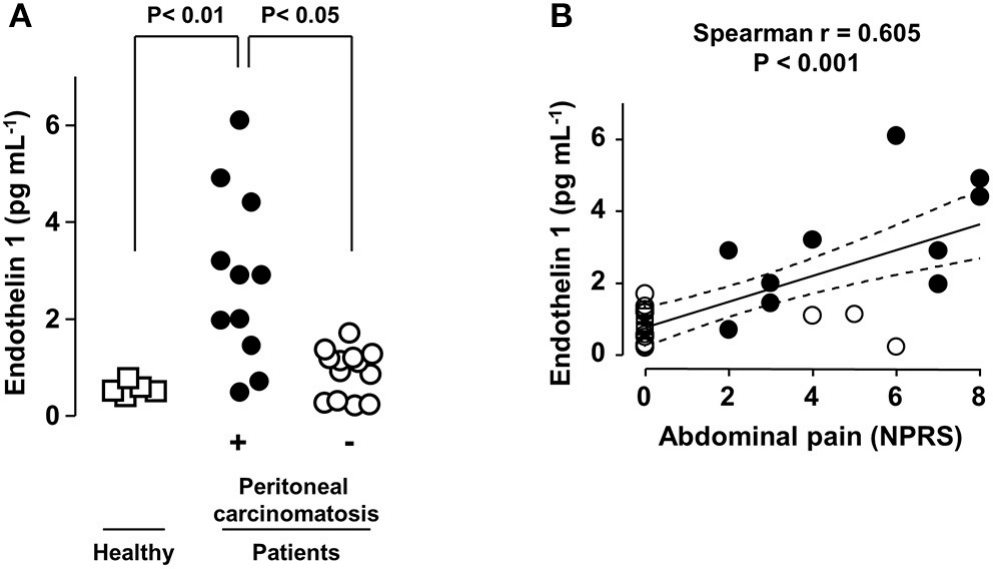
Abdominal pain intensity correlates with plasma EDN1 levels in PC patients. **(A)** Plasma levels of EDN1 in healthy volunteers (□) and in patients with digestive tumors without (◦) or with (•) peritoneal carcinomatosis (PC). Statistical analysis was performed using Kruskal-Wallis test followed by a *post-hoc* Dunn's multiple comparison test. **(B)** Correlation between EDN1 plasma levels and abdominal pain assessed using the Numeric Pain Rating Scale (NPRS: score between 0 and 10) at the time of blood sampling. Each point represents one patient. The strength of the correlation between pain intensity and EDN1 plasma concentration was evaluated using the non-parametric Spearman's correlation coefficient.

A total of 24 patients (nine men and 15 women) and five healthy donors were included in the study. The median age of the patients was 71.5 years (range: 46–90 years). The abdominal tumors in these patients were located in the colon (14 patients), kidney (one patient), ovary (three patients), pancreas (two patients), or stomach (two patients). One patient with esophageal cancer and PC, and one patient with melanoma and PC were also included in the study. Overall, 11 of the patients had PC and five of these patients had undergone nasogastric suction for bowel obstruction. The other 13 patients had local abdominal tumors without PC (Figure 1A).

### EDN1 Is More Abundant in the Plasma of Patients With Painful Digestive Tract Tumors With PC

Ninety-one percent (*n* = 10/11) of the patients with PC suffered from abdominal pain. In contrast, 77% (*n* = 10/13) of the patients without PC presented with no pain (Figure 1B). None of the five healthy donors included in the study reported any abdominal pain.

Analysis of the blood samples collected at the time of abdominal pain scoring revealed that the plasma levels of EDN1 were significantly higher in patients with PC than in the five healthy donors and in the 13 patients with abdominal tumors without PC (Figure 1A). Plasma EDN1 levels below 2 pg.mL^−1^ were rarely associated with significant pain (NPRS > 4). All the patients with EDN1 plasma levels higher than 4 pg mL^−1^, including solely patients suffering from PC, experienced severe (4 < NPRS < 6) to very severe (NPRS > 6) abdominal pain (Figure 1B).

Analysis revealed a positive correlation between the intensity of the abdominal pain and EDN1 concentrations in the plasma in the 24 patients and five healthy controls (correlation coefficient = 0.605; *p* < 0.001). The correlation coefficient increased to 0.7 when only patients with PC were included in the analysis (*p* < 0.01; Figure 1B, black circles).

### EDN1 Induces Visceral Hypersensitivity and Enhanced Colon Contractions in Mice

**Figure 2:**
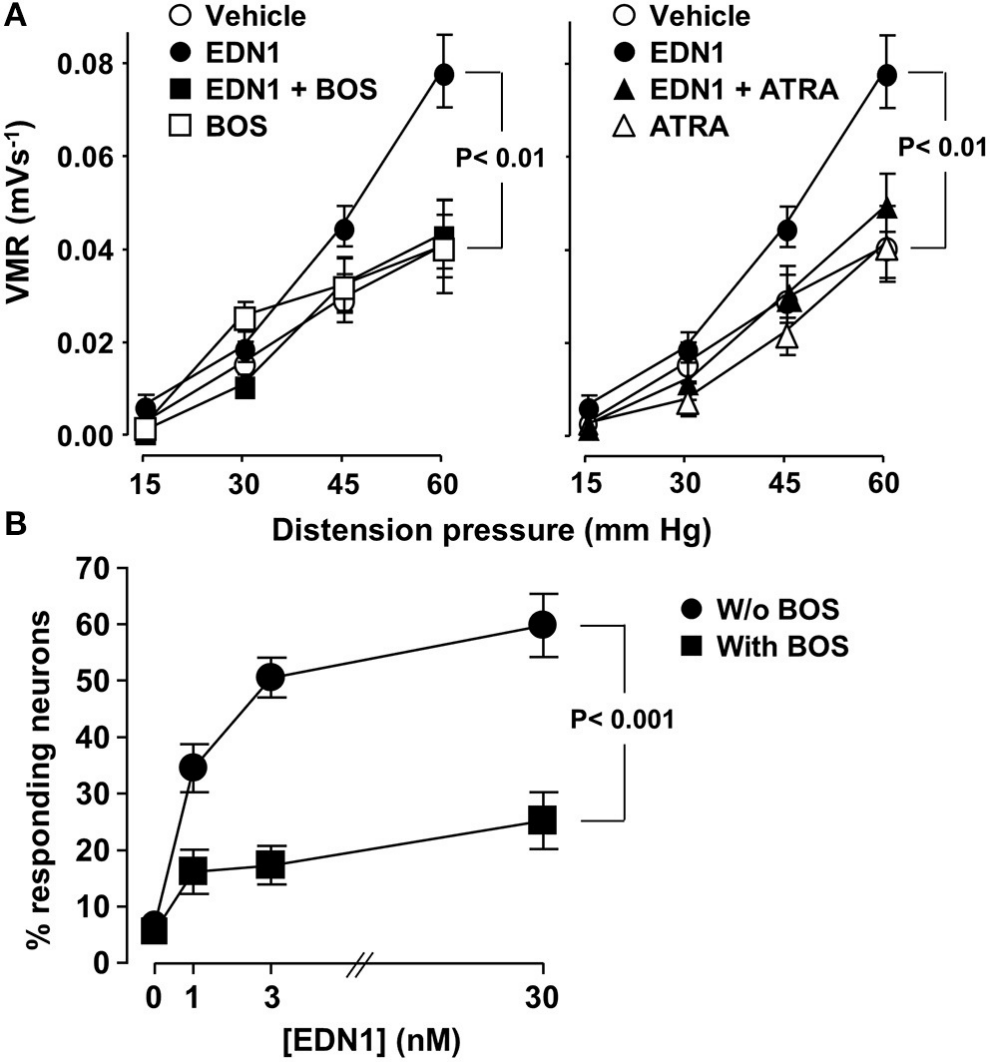
EDN1 induces visceral hypersensitivity *in vivo* and activates sensory neurons *in vitro* in mice. **(A)** Colonic sensitivity was measured in C57Bl/6 mice 30 min after intraperitoneal administration of a vehicle (DMSO, *n* = 19, ◦), EDN1 (*n* = 22, •), BOS (*n* = 5, □), or ATRA (*n* = 8, △). For inhibitory experiments, BOS (■) (left panel) or ATRA (▴) (right panel) was injected 30 min before EDN1 (*n* = 17–18). Visceromotor responses (VRM) were recorded in response to distension pressure of 15, 30, 45, and 60 mmHg. Data are expressed as mean ± SEM. Comparisons with mice injected with the vehicle were analyzed using repeated-measures two-way ANOVA. **(B)** Calcium flux was measured in primary cultures of DRG sensory neurons isolated from C57Bl/6 mice in response to increasing amounts of EDN1 in the absence (•) or in the presence of 10 μM BOS (■). Data presented are the mean ± SEM of the percentage of responding neurons calculated from three independent experiments performed in three wells per condition and 60–80 neurons per well. Statistical analysis was performed using repeated-measures two-way ANOVA.

The role of EDN1 as mediator of visceral hypersensitivity was investigated by evaluating its effects on abdominal muscle contractions in mice. EDN1 significantly enhanced the intensity of abdominal muscle contractions in response to increasing colorectal distension pressure (Figure 2A). This EDN1-induced visceral hypersensitivity was inhibited by exposure to BOS (Figure 2A, left panel), an antagonist of both EDNRs (EDNRA and EDNRB). A similar level of inhibition was observed after exposure to ATRA (Figure 2A, right panel), a specific antagonist of EDNRA, suggesting a predominant contribution of the EDN1/EDNRA signaling pathway in the hypersensitivity response.

The ability of EDN1 to directly activate sensory neurons was then assessed by investigating calcium mobilization in a primary culture of neurons isolated from mouse DRG. Exposure to EDN1 led to calcium mobilization, with the percentage of neurons responding to EDN1 being dose-dependent (Figure 2B). This EDN1-induced calcium mobilization was inhibited by BOS. Exposure to this EDNR antagonist led to a significant reduction in both the number of EDN-responding neurons and the signal intensity of calcium flux [1.6 ± 0.04 (30 nM EDN1) vs. 1.4 ± 0.06 (30 nM EDN1 + BOS), Mann-Whitney *U*-test *p* < 0.01]. Taken together these findings indicate that EDN1 directly triggered neuronal activation *via* its interaction with the EDNRs expressed on the sensory neurons.

**Figure 3:**
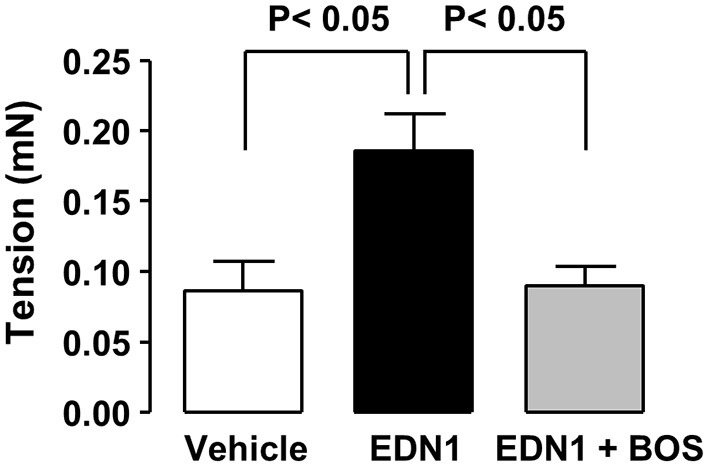
EDN1 increases the amplitude of colonic contractions in mice. Colonic contractions were recorded for 10 min on 10-mm proximal colon segments from C57Bl/6 mice after exposure to the vehicle (DMSO, white bar), EDN1 alone (black bar), or EDN1 plus BOS (gray bar) (*n* = 6–7 mice per group). Colonic contraction amplitudes are expressed in mN. Data are expressed as the mean ± SEM. Statistical analysis was performed using Kruskal-Wallis test followed by a *post-hoc* Dunn's multiple comparison test.

Mice exposed to EDN1 exhibited acute diarrhea (data not shown). This observation led us to examine the effect of EDN1 on intestinal motility. Our *ex vivo* measurements in proximal colon segments revealed that EDN1 exposure led to an increase in the amplitude of colonic contractions (Figure 3). This EDN1-induced alteration in colonic motility was inhibited by BOS (Figure 3).

## Discussion

Our study provided evidence that EDN1 could be a key mediator of pain in PC. Using data obtained from assessments of EDN1 plasma levels and pain severity scores in PC patients, combined with *in vivo, ex vivo*, and *in vitro* experimental data from mice on the effects and mode of action of this peptide, our study suggests that EDN1 could mediate pain in PC by triggering visceral hypersensitivity and alterations in intestinal motility *via* direct activation of the EDNRs to induce calcium ion mobilization and activation of the pain signaling pathway in nociceptive fibers.

The results of our exploratory study involving patients with PC revealed that EDN1 was more abundant in patients suffering from advanced abdominal tumors with PC than in healthy donors or patients with digestive tract tumors without PC. In addition, the concentration of EDN1 in plasma samples correlated with the intensity of abdominal pain, thus indicating that EDN1 could play a key role in the mediation of visceral pain in patients with PC. Indeed, although EDN1 was first described as an endogenous vasoactive peptide [3], it has since been reported to play a key role in the mediation of both somatic and visceral pain [11, 16]. In agreement with the findings of our study of patients with PC, elevated levels of EDN1 in the plasma have also been observed in patients with a number of chronic painful intestinal diseases including Crohn's disease and ulcerative colitis [17, 18]. Correlations between tumor growth, pain and EDN1 plasma levels have also been reported in animal models and patients with orofacial tumors and digestive cancer with spontaneous pain [19–22].

The interesting findings in patients with PC led us to investigate the effect of END1 on abdominal muscle contractions and intestinal motility in mice. In agreement with our findings suggesting that EDN1 plays a role in visceral pain in PC, we showed that EDN1 induced visceral hypersensitivity and increased colonic peristalsis in mice.

As intestinal spasms associated with increased peristaltic activity may induce painful sensations, we investigated whether EDN1 could directly activate the pain signaling pathway by investigating the ability of this peptide to induce calcium mobilization in primary cultures of sensory neurons. Our results showed that exposure to EDN1 led to a dose-dependent induction of calcium mobilization, suggesting that EDN1 directly activates the EDNRs in sensory neurons. This is a potentially important finding as these receptors are expressed all along the pain signaling pathway, including in both C-type and Aδ-type nociceptive fibers [9, 12, 23–27].

We also showed that the effects of EDN1 on abdominal muscle contractions, intestinal motility and calcium ion mobilization observed in our study could be inhibited by EDNR antagonists. Indeed, BOS has already been shown to have an analgesic effect on pain in preclinical animal models of carcinoma [22]. Our inhibition studies also indicated that the EDN1/EDNRA signaling pathway plays the major role in mediating EDN1-induced visceral hypersensitivity. However, the respective contributions of each of the EDNRs to pain and intestinal hypercontractility needs further investigation. In addition, the impact of alterations in wall compliance on the response to colorectal distension needs to be evaluated further; although these two processes have been shown to be independent in EDN3-deficient mice [16].

Although more extensive studies are required, our results provide strong evidence for a major role for EDN1 in the mediation of pain in patients with abdominal tumors and PC. These findings may form the basis for larger therapeutic trials investigating the clinical use of EDNR antagonists to alleviate refractory visceral pain in patients with PC.

## Data Availability Statement

The raw data supporting the conclusions of this article will be made available by the authors, without undue reservation.

## Ethics Statement

The studies involving human participants were reviewed and approved by Ethic Committee of the Sud-Seine et Marne Hospital (05/12/2015). The patients/participants provided their written informed consent to participate in this study. This animal study was reviewed and approved by the Animal Care and Ethics Committee of US006/CREFE (CEEA-122; application number APAFIS #7762- CE2016112509278235V2).

## Author Contributions

CG, BD, SC, and AL enrolled patients. LB, CD, AF, CK, and NC designed and conducted experiments and acquired and analyzed the data. LL and Y-YZ acquired data. CG, CB, and GD supervised the study, designed experiments, analyzed data, and wrote the manuscript. All authors contributed to the article and approved the submitted version.

## Conflict of Interest

The authors declare that the research was conducted in the absence of any commercial or financial relationships that could be construed as a potential conflict of interest.
